# A Nomogram for Predicting the Need of Postoperative Tracheostomy in Patients With Aneurysmal Subarachnoid Hemorrhage

**DOI:** 10.3389/fneur.2021.711468

**Published:** 2021-08-27

**Authors:** Xiao-Yong Chen, Yue Chen, Ni Lin, Jin-Yuan Chen, Chen-Yu Ding, De-Zhi Kang, Deng-Liang Wang, Wen-Hua Fang

**Affiliations:** ^1^Department of Neurosurgery, Neurosurgical Research Institute, The First Affiliated Hospital, Fujian Medical University, Fuzhou, China; ^2^The School of Medical Technology and Engineering, Fujian Medical University, Fuzhou, China; ^3^Department of Ophthalmology, The First Affiliated Hospital of Fujian Medical University, Fuzhou, China; ^4^Fujian Key Laboratory of Precision Medicine for Cancer, The First Affiliated Hospital, Fujian Medical University, Fuzhou, China; ^5^Key Laboratory of Fujian Higher Education Institutions, The First Affiliated Hospital, Fujian Medical University, Fuzhou, China

**Keywords:** nomogram, predict, tracheostomy, aneurysmal subarachnoid hemorrhage, need

## Abstract

**Objective:** Early identification for the need of tracheostomy (TT) in aneurysmal subarachnoid hemorrhage (aSAH) patients remains one of the main challenges in clinical practice. Our study aimed to establish and validate a nomogram model for predicting postoperative TT in aSAH patients.

**Methods:** Patients with aSAH receiving active treatment (interventional embolization or clipping) in our institution between June 2012 and December 2018 were retrospectively included. The effects of patients' baseline information, aneurysm features, and surgical factors on the occurrence of postoperative TT were investigated for establishing a nomogram in the training cohort with 393 patients. External validation for the nomogram was performed in the validation cohort with 242 patients.

**Results:** After multivariate analysis, higher age, high neutrophil-to-lymphocyte ratio (NLR), high World Federation of Neurological Surgeons Scale (WFNS), and high Barrow Neurological Institute (BNI) grade were left in the final logistic regression model. The predictive power of the model was excellent in both training cohort and validation cohort [area under the curve (AUC): 0.924, 95% confidence interval [CI]: 0.893–0.948; AUC: 0.881, 95% CI: 0.833–0.919]. A nomogram consisting of these factors had a C-index of 0.924 (95% CI: 0.869–0.979) in the training cohort and was validated in the validation cohort (C-index: 0.881, 95% CI: 0.812–0.950). The calibration curves suggested good match between prediction and observation in both training and validation cohorts.

**Conclusion:** Our study established and validated a nomogram model for predicting postoperative TT in aSAH patients.

## Introduction

Aneurysmal subarachnoid hemorrhage (aSAH) is an acute cerebrovascular disease which causes serious damage to the central nervous system and pathophysiological consequences on many organs of the body (1). A ruptured cerebral aneurysm is the most common cause of spontaneous subarachnoid hemorrhage (SAH) with high mortality rates, accounting for 85% of spontaneous (SAH) with a 40–50% case fatality rate (2). For aSAH patients, a standard airway management following a systematic airway assessment, including endotracheal intubation and tracheostomy (TT), is a crucial part of the whole treatment process.

As a common and effective airway management strategy, TT could reduce airway resistance and improve airway compliance, resulting in reduction of the use of sedatives and respiratory complications especially in those patients requiring prolonged mechanical ventilation. In patients with hemorrhagic stroke, the rates of TT have been found to be significantly higher than in non-neurologic patients (3). Also, several retrospective studies confirmed that the timing of TT could have significant effects in outcomes of patients with hemorrhagic stroke (4, 5). Previously, the feasibility, safety, and reduction of sedation need of early TT in ventilated stroke patients have been confirmed in a randomized pilot trial (6). Recently, a retrospective study stated that 31% of patients with hemorrhagic stroke underwent TT and SAH was confirmed to be a risk factor associated with increased likelihood of TT (7). In addition, earlier TT was proved to make a significant contribution to shorter overall hospitalization in this study (7).

Considering the potential benefits of timely TT, there is a great necessity to identify patients who need TT as early as possible. However, early identification for the need of TT remains one of the main challenges in clinical practice. The stroke-related Early Tracheostomy score (SETscore) was previously used as a screening tool in the pilot trial Stroke-related Early Tracheostomy vs. Prolonged Orotracheal Intubation in Neurocritical care Trial 2 (SETPOINT2) for trial inclusion (8). In several studies, this scoring scale for estimating the 2-week ventilation need has been confirmed in predicting TT need for stroke patients and showed moderate predictive performance (9, 10). However, SETscore was not specially used to predict TT need in aSAH patients. Therefore, our study retrospectively investigated the effects of patients' baseline information, aneurysm features, and surgical factors on the occurrence of postoperative TT, aiming to establish and validate a nomogram model for predicting postoperative TT in aSAH patients.

## Materials and Methods

### Study Population

**Figure 1:**
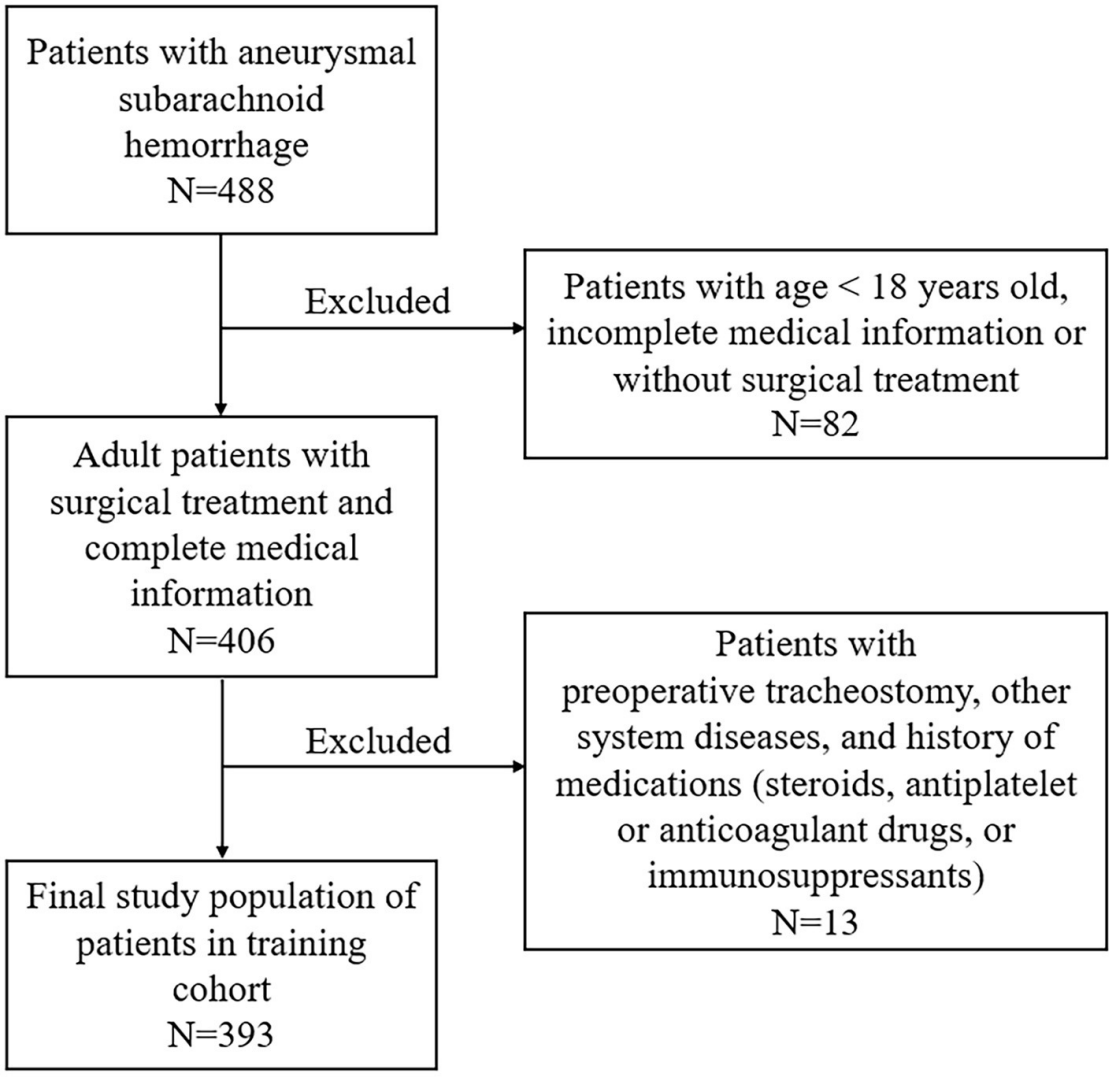
Flowchart of aneurysmal subarachnoid hemorrhage patient selection in the training cohort.

This study was performed at the First Affiliated Hospital of Fujian Medical University. It was designed in accordance with the guidelines outlined in the Declaration of Helsinki and approved by the local ethics committee of the First Affiliated Hospital of Fujian Medical University. The requirement of informed consent was waived due to its retrospective design. Patients with aSAH admitted to our institution for receiving active treatment (interventional embolization or clipping) between June 2012 and December 2018 were retrospectively included. Possible related factors for predicting postoperative TT in 393 patients admitted between June 2012 and June 2016 were identified for establishing a nomogram (training cohort, Figure 1). External validation was performed in a validation cohort consisting of 242 patients admitted between July 2016 and December 2018. The inclusion criteria are as follows: (1) age >18 years old; (2) a diagnosis of aSAH based on preoperative computed tomography angiography (CTA) or digital subtraction angiography (DSA); (3) received surgical treatment including interventional embolization or clipping after admission; (4) patients admitted within 48 h and did not receive intervention before admission; (5) preoperative routine blood test was obtained in emergency room or inpatient wards within the first 2 h of admission; and (6) other medical records were complete. Exclusion criteria were as follows: (1) patients were <18 years old; (2) incomplete medical information; (3) patients with previous or preoperative TT; (3) previous use of steroids, antiplatelet or anticoagulant drugs, or immunosuppressants; and (4) combined other neurological diseases or serious diseases.

### Patient Management

The diagnosis and treatment of aSAH patients were implemented based on the guidelines for the management of aSAH (11). All patients underwent surgery to manage the ruptured aneurysms. After evaluating the neurological function, airway, age, and other factors, the decision of postoperative TT was jointly made by neurosurgeons, intensive care unit clinicians, and otorhinolaryngologists for those patients who required artificial airway and respiratory support more than 2 weeks.

### Clinical, Imaging, and Laboratory Variables

Patient data were collected, including general demographics, history of present illness, past medical history, neurological status on admission, imaging data, treatment regimens, surgical information, postoperative TT, and other related data. All patients underwent a CTA or digital subtract angiography (DSA) before surgery to determine the diagnosis and aneurysm features. The aneurysm features were assessed by neurosurgeons based on CTA or DSA. Patients were categorized into lower age group (age <60 years) and higher age group (age ≥ 60 years). In addition, the cutoff values were 151 mmHg for admission systolic blood pressure (SBP) and 3.0 h for operation time (Table 1).

**Table 1:** The cutoff value and area under the curve of the possible predictive factors of postoperative tracheostomy in training cohort.

**Parameter**	**Cutoff value**	**AUC**	**Sensitivity (%)**	**Specificity (%)**	**Youden index**	**95% CI of AUC**
Admission SBP, mmHg	151	0.647	58.97	70.34	0.293	0.548–0.647
NLR	11.03	0.755	76.92	74.01	0.509	0.734–0.819
PLR	168	0.606	76.92	44.35	0.213	0.559–0.658
LMR	1.56	0.697	58.97	83.05	0.420	0.656–0.748
SIRI	9.27	0.698	48.72	91.24	0.400	0.675–0.766
Operation time, h	3.0	0.629	53.85	72.03	0.259	0.555–0.654

*AUC, area under curve; CI, confidence interval; SBP, systolic blood pressure; NLR, neutrophil-to-lymphocyte ratio; PLR, platelet-to-lymphocyte ratio; LMR, lymphocyte–monocyte ratio; SIRI, systemic inflammatory response index*.

Delayed cerebral vasospasm (DCVS) was defined as a sustained constriction of cerebral blood vessels after aSAH which is related to delayed neurological deficit of the corresponding region (12). Delayed cerebral ischemia (DCI) was defined as clinical deterioration caused by DCI and/or presence of cerebral infarction on imaging examination within 6 weeks after SAH (13).

Neurological status (14–19) was evaluated on admission by the responsible neurosurgeons, including World Federation of Neurological Surgeons Scale (WFNS) (14), modified Fisher (mFisher) grade (17), and Barrow Neurological Institute (BNI) grading scale (19). To simplify the final model and minimize the assessor bias, the neurological status scales were categorized into two grades: low and high WFNS grade (grades 1–2 and grades 3–5); low and high mFisher grade (grades 0–2 and grades 3–4); and low and high BNI grade (grades 1–3 and grades 4–5).

Before surgery, all patients underwent general preoperative blood tests according to standard laboratory test procedures in emergency room or neurosurgical wards within 2 h of admission. Based on the routine peripheral blood tests, inflammatory markers (20, 21) including neutrophil-to-lymphocyte ratio (NLR), platelet-to-lymphocyte ratio (PLR), lymphocyte–monocyte ratio (LMR), and SIRI (systemic inflammatory response index) were also included in our study. NLR = neutrophil/lymphocyte, PLR = platelet/lymphocyte, LMR = lymphocyte/monocyte, SIRI = monocyte × neutrophil/lymphocyte. The cutoff values were 11.03 for NLR, 168 for PLR, 1.56 for LMR, and 9.27 for SIRI (Table 1). High values of NLR, PLR, and SIRI and low values of LMR were considered to reflect the “inflammatory state” of patients (20–22).

### Buildup of the Model and the Validation of the Nomogram

The potential risk factors of TT in the univariable analysis were used to establish the models. Considering the similarity of mFisher grade and BNI grading scale in reflecting blood clot burden, we established two models with mFisher grade or BNI grading scale and compared their predictive power. The other factors together with mFisher grade or BNI grading scale in the models were all significantly associated with TT in the multivariable analysis. The model with better predictive performance in both training cohort and validation cohort was selected to establish a nomogram. The performance and consistency of the nomogram was validated by the validation cohort.

### Statistical Analysis

Statistical analysis was performed using SPSS 19.0 statistical software (SPSS, Inc., Chicago, IL, USA) and R statistical software (R version 4.0.3, R Project, www.r-project.org). *p* < 0.05 was considered statistically significant. Continuous variables, presented as mean ± standard deviation, were analyzed by the two-sample *t* test. They were expressed as median (interquartile range) and analyzed by non-parametric test if they did not meet normal distribution. Categorical variables, described as frequency (percentage), were analyzed using the χ^2^-test or Fisher exact test. All available baseline, imaging, and surgical variables were included in univariable logistic regression analysis to determine their association with postoperative TT. Variables which had a univariable association of *p* < 0.10 were included for multivariable logistic regression analysis. Forward stepwise multivariable regression was performed to create the final model with *p* < 0.05. The predictive values of models were evaluated by receiver operating characteristic (ROC) curve analysis and presented with area under the curve (AUC), sensitivity, specificity, and *Youden* index. DeLong's test was used to assess and compare the predictive power of the models. Decision curve analyses (DCA), integrated discrimination improvements (IDI), and Net Reclassification Index (NRI) were also mapped and calculated to compare the clinical utility of different models.

The model with better predictive performance in the training cohort with 393 patients was validated by a validation cohort with 242 patients according to ROC analysis, DCA curve, NRI, and IDI. Then, the model was selected to establish a nomogram for patients with postoperative TT. The Harrell concordance index (C-index) and a calibration curve were utilized to validate the performance of the nomogram in both training cohort and validation cohort. Bootstrapping with 1,000 samples was performed to measure internal calibration and the discrimination in both training cohort and validation cohort. A C-index over 0.7 indicates a good nomogram model.

## Results

### Patients' Characteristics

Among the patients with TT, the median time from aSAH onset to TT placement was 9.8 days (7.0–15.1 days). Patients with TT had a significantly higher risk for DCVS and DCI than those without TT (*p* < 0.001, *p* = 0.003). Both high mFisher grade and high BNI grade were associated with higher risk of DCVS (*p* = 0.004, *p* < 0.001) and DCI (*p* = 0.002, *p* < 0.001).

The demographics and clinical variables of the training and validation cohorts are presented in Table 2. Of the 393 patients in the training cohort, 39 patients were divided into a TT group and 354 patients were divided into a non-TT group. In the training cohort, 28.2% (111 of 393) of the enrolled patients belonged to the higher age group; 39.4% (155 of 393) of the enrolled patients were male. Age, hypertension, admission SBP, NLR, PLR, LMR, SIRI, and operation time were significantly different between the two groups (Table 2).

**Table 2:** The demographic and clinical variables of training and validation cohorts.

**Training cohort**				**Validation cohort**			
**Parameter**	**TT**	**Non-TT**	***p*-value**	**Parameter**	**TT**	**Non-TT**	***p*-value**
***N***	39	354		**N**	25	217	
**Baseline information**				**Baseline information**			
Age ≥60 years	19 (17.1%)	92 (82.9%)		Age ≥60 years	15 (17.0%)	73 (83.0%)	
Male	20 (12.9%)	135 (87.1%)		Male	9 (10.0%)	81 (90.0%)	
Hypertension	25 (16.6%)	126 (83.4%)		Hypertension	15 (13.8%)	94 (86.2%)	
Diabetes mellitus	4 (23.5%)	13 (76.5%)		Diabetes mellitus	1 (9.1%)	10 (90.9%)	
Stroke history	0 (0%)	3 (100.0%)		Stroke history	0 (0.0%)	2 (100.0%)	
Smoking	3 (8.8%)	31 (91.2%)		Smoking	1 (5.6%)	17 (94.4%)	
Admission SBP, mmHg	155 (134–166)	140 (126–159)	0.045	Admission SBP, mmHg	154 (136–170)	144 (126–161)	0.047
Admission DBP, mmHg	86 (78–100)	82 (76–93)	0.121	Admission DBP, mmHg	86 (75–94)	85 (75–98)	0.528
Time from onset to admission, h	18 (6–36)	24 (8–96)	0.110	Time from onset to admission, h	13 (6–48)	17 (7–48)	0.586
NLR	15.8 (11.1–19.7)	7.0 (4.1–11.4)	<0.001	NLR	15.5 (10.4–23.7)	9.3 (5.9–13.9)	<0.001
PLR	230.8 (168.1–302.2)	179.9 (129.8–248.4)	0.025	PLR	233.3 (181.5–317.3)	203.0 (143.8–286.3)	0.104
LMR	1.5 (1.1–2.9)	2.8 (1.9–3.9)	<0.001	LMR	1.3 (1.0–2.6)	2.3 (1.6–3.5)	0.002
SIRI	8.2 (3.6–14.0)	3.1 (1.7–5.4)	<0.001	SIRI	9.9 (4.2–14.6)	4.1 (2.3–6.7)	<0.001
WFNS grade 3–5	32 (44.4%)	40 (55.6%)		WFNS grade 3–5	20 (29.9%)	47 (70.1%)	
mFisher score 3–4	31 (37.8%)	51 (62.2%)		mFisher score 3–4	19 (31.1%)	42 (68.9%)	
BNI grade 4–5	31 (44.3%)	39 (55.7%)		BNI grade 4–5	20 (28.6%)	50 (71.4%)	
**Aneurysm features and surgical factors**				**Aneurysm features and surgical factors**			
Time from admission to surgery, h	45 (10–96)	55 (26–81)	0.215	Time from admission to surgery, h	48 (26–93)	63 (38–92)	0.535
Aneurysm ≥5 mm	31 (12.4%)	218 (87.6%)		Aneurysm ≥5 mm	15 (13.2%)	99 (86.8%)	
Posterior circulation aneurysm	13 (13.3%)	85 (86.7%)		Posterior circulation aneurysm	9 (10.5%)	77 (89.5%)	
Single aneurysm	28 (9.3%)	272 (90.7%)		Single aneurysm	22 (12.4%)	156 (87.6%)	
Surgical clipping	29 (9.6%)	274 (90.4%)		Surgical clipping	22 (11.5%)	170 (88.5%)	
Operation time, h	3.5 (2.5–4.0)	3.0 (2.0–3.5)	0.029	Operation time, h	3.0 (2.8–4.0)	2.5 (9.3–3.5)	0.074

*Values are reported as number, number (%), and median (25–75%). TT, tracheostomy; SBP, systolic blood pressure; DBP, diastolic blood pressure; NLR, neutrophil-to-lymphocyte ratio; PLR, platelet-to-lymphocyte ratio; LMR, lymphocyte–monocyte ratio; SIRI, systemic inflammatory response index; WFNS, World Federation of Neurological Surgeons Scale; mFisher, modified Fisher score; BNI, Barrow Neurological Institute*.

### The Cutoff Value and Area Under the Curve of the Possible Predictive Factors in the Training Cohort

Table 1 shows that admission SBP = 151 mmHg, NLR = 11.03, PLR = 168, LMR = 1.56, SIRI = 9.27, and operation time = 3.0 h (from 1.0 to 8.0 h) were the optimal cutoff values. The AUC of admission SBP, NLR, PLR, LMR, SIRI, and operation time were 0.647, 0.755, 0.606, 0.697, 0.698, and 0.629, respectively. Based on the cutoff values, the sensitivities of admission SBP, NLR, PLR, LMR, SIRI, and operation time were 58.97, 76.92, 58.97, 48.72, and 53.85%, respectively, and the specificities were 70.34, 74.01, 44.35, 83.05, 91.24, and 72.03%, respectively.

### Association Between Variables and Postoperative TT in aSAH Patients and Establishment of Two Models

Parameters with a significant univariable association (*p* < 0.10) for postoperative TT are presented in Table 3, including age, hypertension, admission SBP, NLR, PLR, LMR, SIRI, GCS, HH grade, WFNS grade, mFisher score, aneurysm size, and operation time. Based on multivariable analysis, two predictive models with mFisher grade or BNI grading scale were produced to explore the best connection among those risk factors. Model A consisted of four independent risk factors, including age ≥60 years (odds ratio [OR] = 3.79, 95% confidence interval [CI]= 1.56–9.44, *p* = 0.004), high NLR (OR = 3.26, 95%CI = 1.24–8.60, *p* = 0.017), high WFNS grade (OR = 7.91, 95%CI = 2.62–23.84, *p* < 0.001), and high mFisher grade (OR = 5.95, 95%CI = 2.05–17.26, *p* < 0.001). Model B also consisted of four independent risk factors, including age ≥60 years (OR = 4.82, 95%CI = 1.79–13.01, *p* = 0.002), high NLR (OR = 3.77, 95%CI = 1.35–10.55, *p* = 0.011), high WFNS grade (OR = 7.09, 95%CI = 2.41–20.88, *p* < 0.001), and high BNI grade (OR = 11.91, 95%CI = 4.17–33.97, *p* < 0.001). The details of the two models are presented in Table 4.

**Table 3:** Univariable logistic regression analysis of postoperative tracheostomy with possible predictive factors in training cohort.

**Parameters**	**OR**	**95% CI**	***p*-value**
Higher age	2.71	1.38–5.29	0.004
Hypertension	3.23	1.62–6.44	0.001
High admission SBP, mmHg	3.41	1.73–6.71	<0.001
High NLR	9.49	4.34–20.75	<0.001
High PLR	2.66	1.23–5.76	0.013
Low LMR	6.34	3.18–12.66	<0.001
High SIRI	9.56	4.63–19.75	<0.001
High WFNS grade	35.89	14.86–86.65	<0.001
High mFisher score	23.02	10.02–52.90	<0.001
High BNI grade	31.30	13.44–72.89	<0.001
Large aneurysm	2.42	1.08–5.41	0.032
Long operation time	3.01	1.54–5.88	0.001

*OR, odds ratio; CI, confidence interval; SBP, systolic blood pressure; NLR, neutrophil-to-lymphocyte ratio; PLR, platelet-to-lymphocyte ratio; LMR, lymphocyte–monocyte ratio; SIRI, systemic inflammatory response index; WFNS, World Federation of Neurological Surgeons Scale; mFisher, modified Fisher score; BNI, Barrow Neurological Institute*.

**Table 4:** Potential factors associated with postoperative tracheostomy by logistic regression.

	**Model A**				**Model B**		
	**OR**	**95% CI**	***p*-value**		**OR**	**95% CI**	***p*-value**
Higher age	3.79	1.52–9.44	0.004	Higher age	4.82	1.79–13.01	0.002
High NLR	3.26	1.24–8.60	0.017	High NLR	3.77	1.35–10.55	0.011
High WFNS grade	7.91	2.62–23.84	<0.001	High WFNS grade	7.09	2.41–20.88	<0.001
High mFisher score	5.95	2.05–17.26	0.001	High BNI grade	11.91	4.17–33.97	<0.001

*OR, odds ratio; CI, confidence interval; NLR, neutrophil-to-lymphocyte ratio; WFNS, World Federation of Neurological Surgeons Scale; mFisher, modified Fisher score; BNI, Barrow Neurological Institute*.

### Comparison of the Predictive Powers of the Two Different Models in the Training and Validation Cohorts

**Figure 2:**
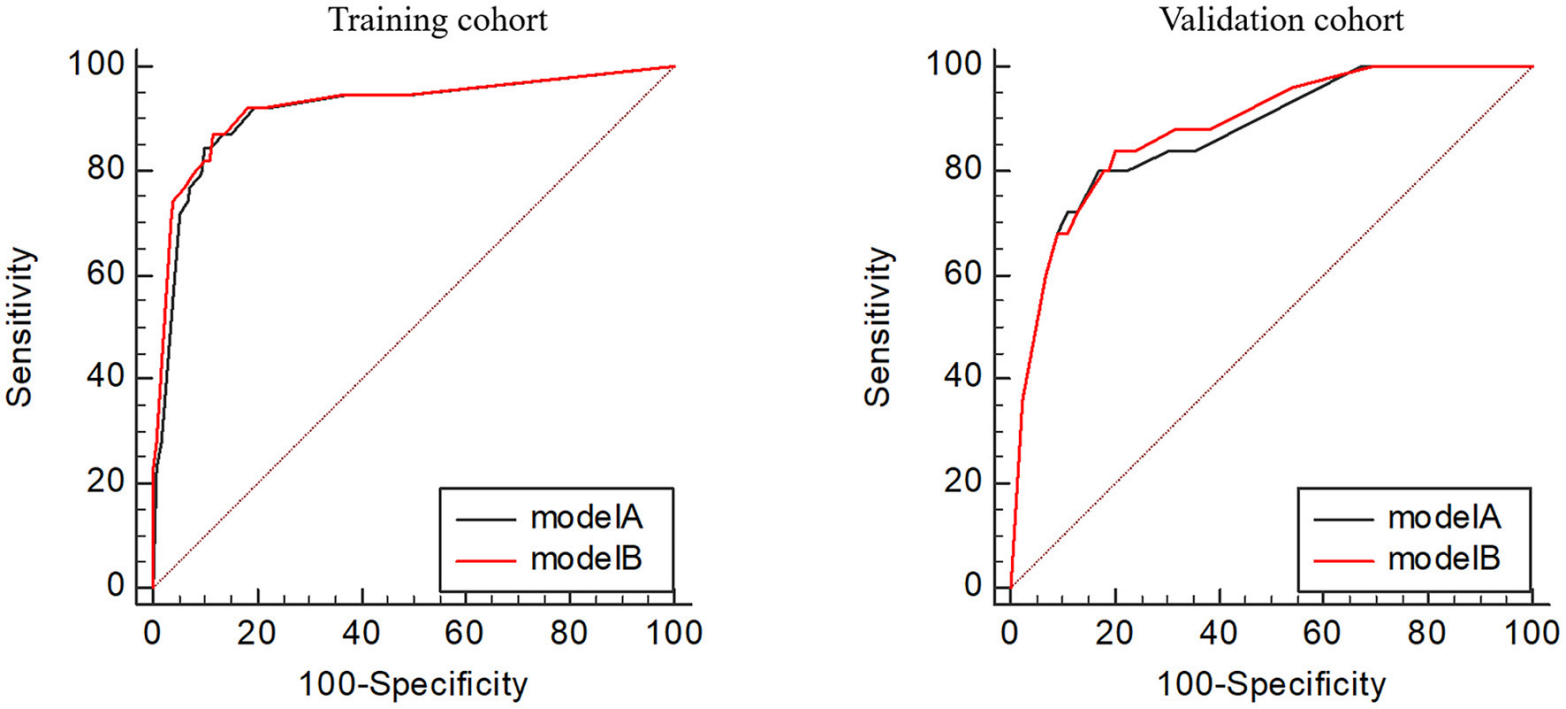
Receiver operating characteristic curves of the two models in the training and validation cohorts.

**Figure 3:**
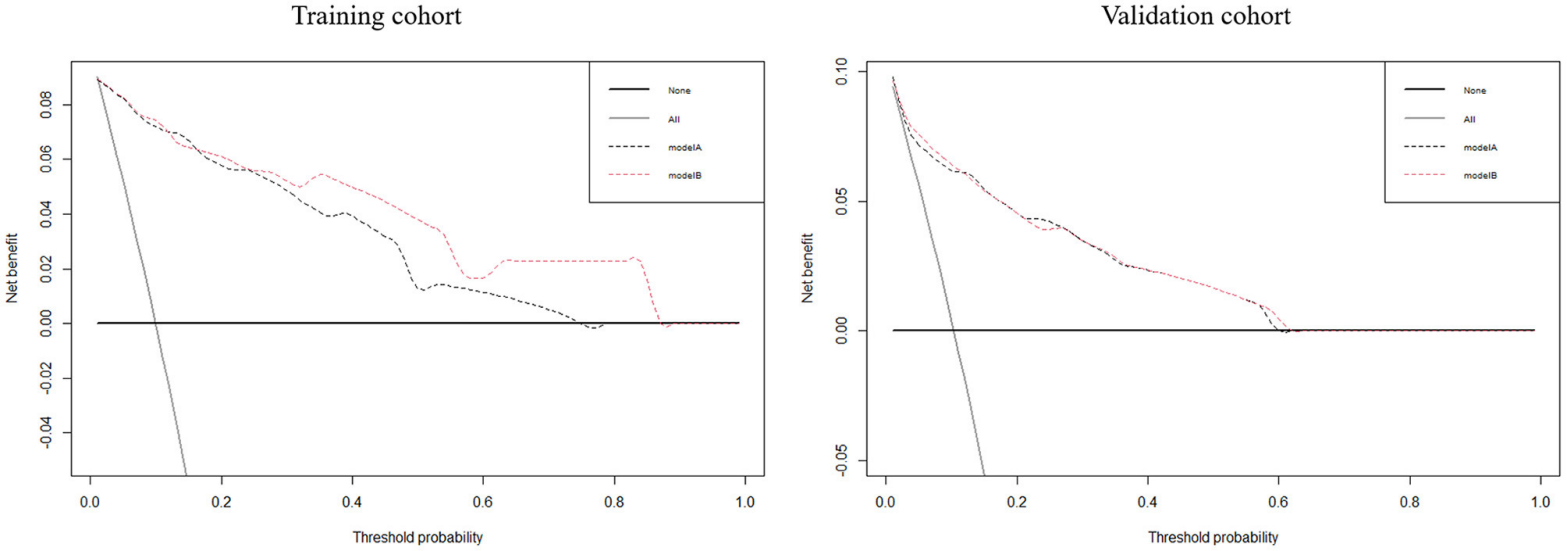
The decision curve analyses for the two models to predict the occurrence of postoperative tracheostomy in aneurysmal subarachnoid hemorrhage patients in the training and validation cohorts.

Utilizing ROC analysis, the predictive powers of model A and model B in the training and validation cohorts are presented in Figure 2. In the training cohort, the predictive power of model A was represented with AUC = 0.914 (95%CI = 0.882–0.940), sensitivity = 84.6%, specificity = 90.1%, and *Youden* index = 0.747; the predictive performance of model B was represented with AUC = 0.924, 95%CI = 0.893–0.948, sensitivity = 87.2%, specificity = 88.4%, and *Youden* index = 0.756. DeLong's test indicated that the AUC of model B was significantly higher than that of model A (Z = 2.333, *p* = 0.020). Figure 2 shows DCA curves of the two models for predicting postoperative TT. The net benefit afforded by model B was higher between the threshold probabilities of 0.2 and 0.5. To further compare the performance of the two models, NRIs and IDIs were calculated and are shown in Figure 3. Compared to model A, the NRI and IDI approaches indicated that the predictive power of model B was better (NRI = 0.06, 95%CI = −0.01–0.13, *p* = 0.10; IDI = 0.07, 95% CI = 0.05–0.08, *p* < 0.01).

**Figure 4:**
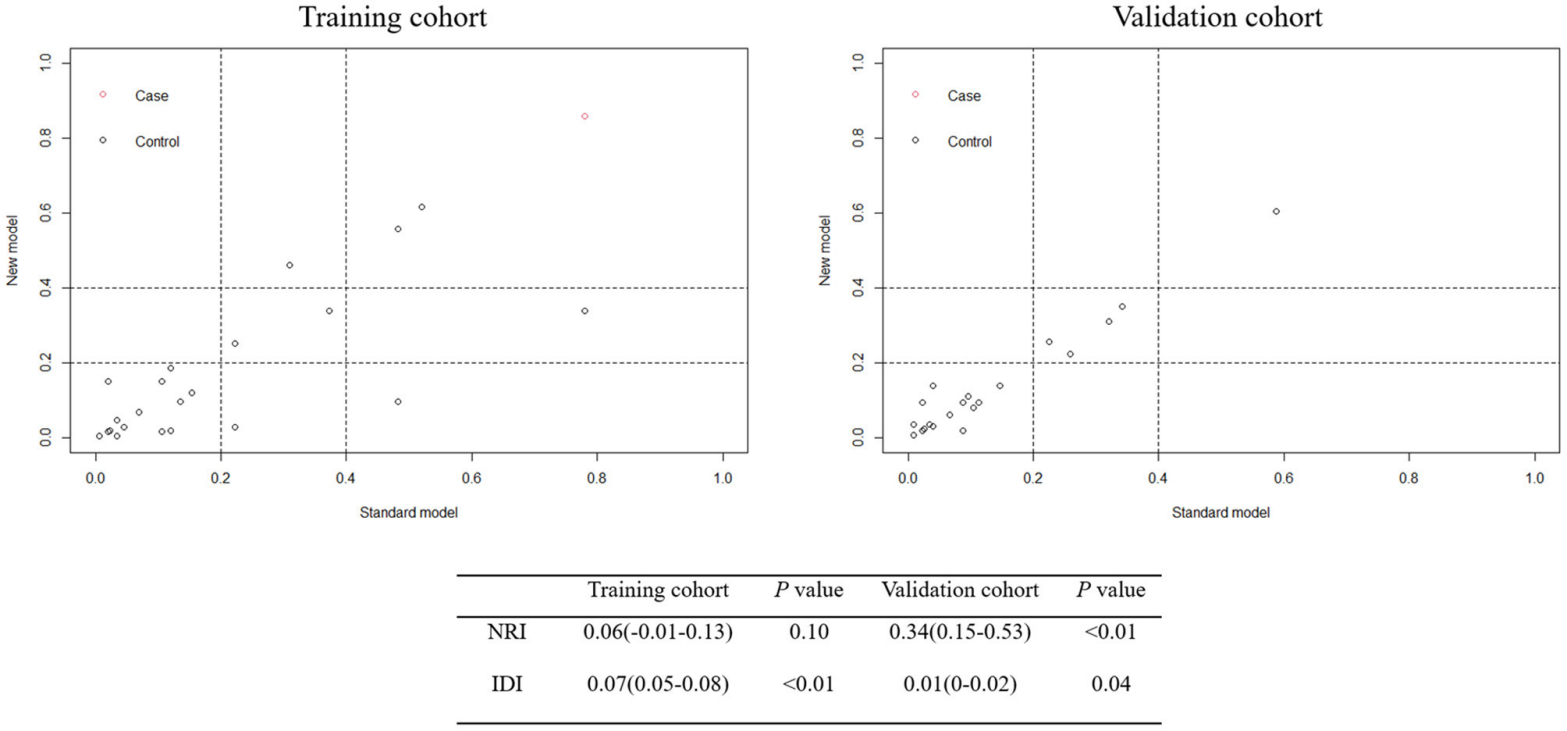
Net Reclassification Index (NRI) and Integrated Discrimination Improvement (IDI) between model A and model B in the training and validation cohorts.

In the validation cohort, the predictive power of model A was represented with AUC = 0.870 (95%CI = 0.821–0.909), sensitivity = 80.0%, specificity = 83.0%, and *Youden* index = 0.630; the predictive performance of model B was represented with AUC = 0.881, 95%CI = 0.833–0.919, sensitivity = 84.0%, specificity = 79.7%, and *Youden* index = 0.637. DeLong's test indicated that the AUC of model B was higher than that of model A, but not statistically significant (Z = 0.671, *p* = 0.502). Figure 3 shows the DCA curves of the two models for predicting postoperative TT. The net benefit afforded by model B was higher between the two models. Compared to model A (Figure 4), the NRI and IDI approaches indicated that the predictive power of model B was better (NRI = 0.34, 95%CI = 0.15–0.53, *p* < 0.01; IDI = 0.01, 95%CI = 0–0.02, *p* = 0.04). Therefore, model B with the BNI grading scale was selected to establish a nomogram for patients with postoperative TT.

### Establishment and Verification of Nomogram

**Figure 5:**
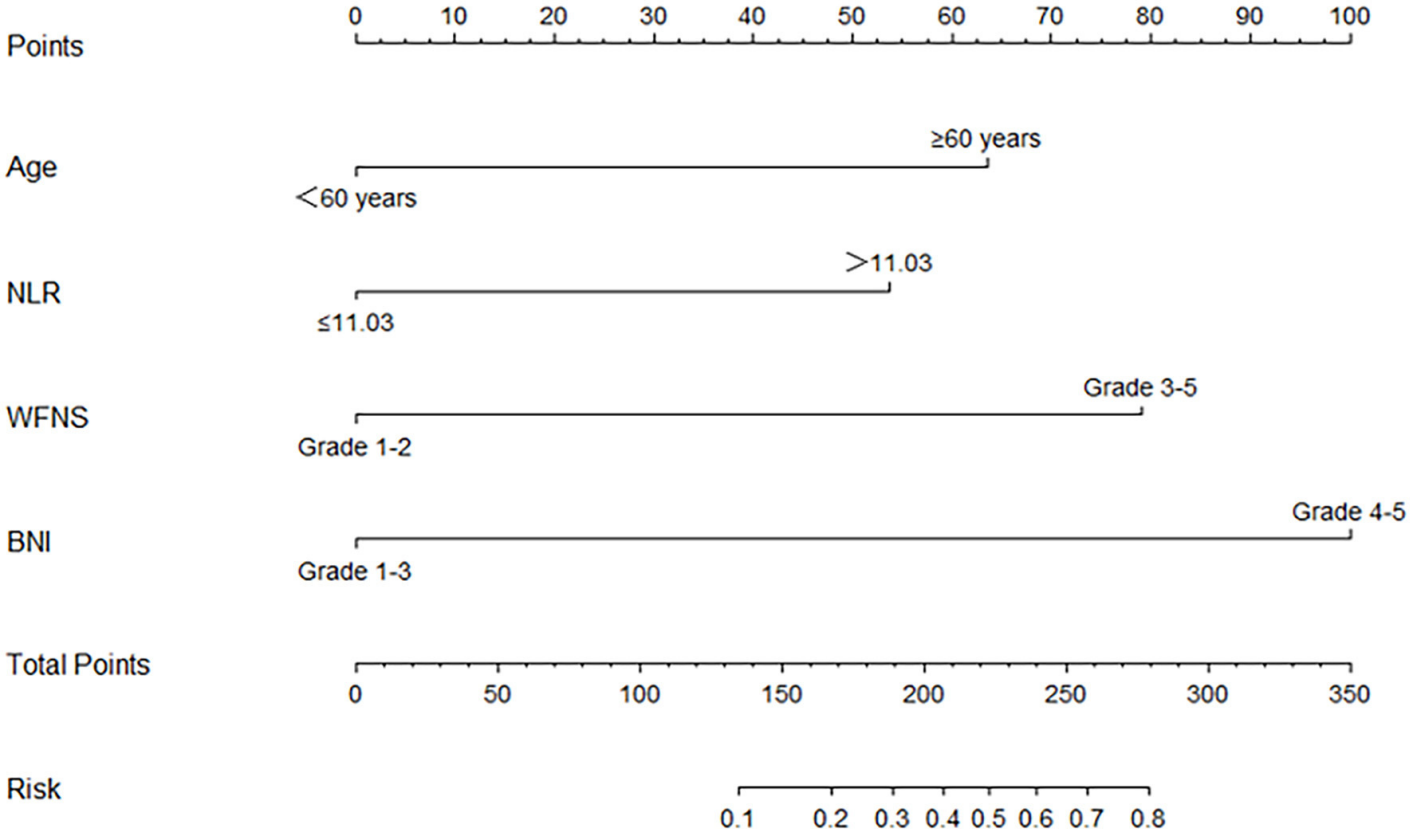
The nomogram for predict the occurrence of postoperative tracheostomy. NLR, neutrophil-to-lymphocyte ratio; WFNS, World Federation of Neurological Surgeons Scale; BNI, Barrow Neurological Institute.

**Figure 6:**
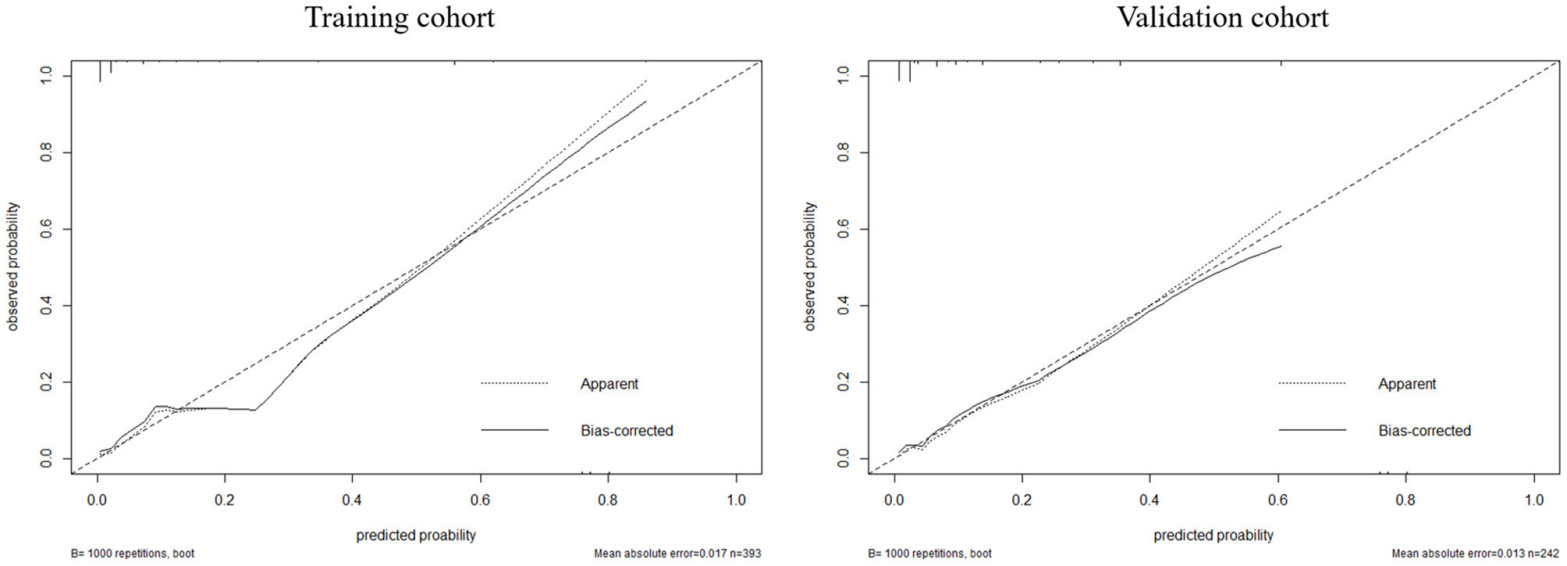
The calibration between the predicted and observed results in the training and validation cohorts.

According to the result of the multivariable logistic regression analysis and comparison of the predictive powers between the two models, age, NLR, WFNS grade, and BNI grade were used as predictors to develop a nomogram model for predicting postoperative TT in aSAH patients (Figure 5). By adding the point of each part in the nomogram model, a sum-up total point was projected on the bottom scale which indicated the risk of postoperative TT. The C-index of the nomogram for predicting postoperative TT was 0.924 (95% CI: 0.869–0.979). The calibration curve of the nomogram suggested a good match between prediction and observation for postoperative TT (Figure 6). Independent validation was performed in the validation cohort, and the C-index was 0.881 (95% CI: 0.812–0.950). A good match was also observed in this cohort for predicting postoperative TT (Figure 6).

## Discussion

Neurosurgical patients are often accompanied by consciousness disturbance in different degrees with the use of sedatives. A significant proportion of these patients need long-term mechanical ventilation. Tracheotomy is usually selected when extubation fails or extubation is not feasible. However, how to judge the success and feasibility of extubation is also full of challenges and disputes. The classic criteria of extubation are less than satisfactory in stroke patients with a high failure rate approaching 37% (23). Several studies have focused on the potential benefits and safety of TT in patients with hemorrhagic stroke. Chen et al. investigated 425 patients with hemorrhagic stroke and concluded that early TT in those patients could reduce hospital costs and hospital stays with no increase in hospital mortality (4). In another study, Ding et al. claimed that early TT could improve the prognosis at 30 days in patients with brainstem hemorrhage (5). However, there are few studies focusing on postoperative TT in aSAH patients specifically (24). The optimal timing and predictive factors of TT in patients with aSAH remain uncertain. Compared with those patients without TT, patients with TT tend to develop poor outcomes due to the greater degree of severity of the disease. Early prediction and identification of patients who need TT could help to provide respiratory support earlier and may improve the prognosis. Therefore, considering the potential benefits of TT, it would be necessary to identify the risk factors and need of TT.

The BNI grade and mFisher grade both reflected the blood clot burden of aSAH patients. Considering their similarity, two models with mFisher grade or BNI grade were produced based on the multivariable analysis. Three risk factors existed in both models, including age ≥60 years, high NLR, and high WFNS grade which remained significant and independent of postoperative TT after adjusting for confounders. We utilized ROC analysis, DCA curves, NRIs, and IDIs to compare the predictive powers of the two models and determined that the model with high BNI grade presented a better predictive performance than another with a high mFisher grade in the training and validation cohorts. Therefore, we selected the model with high BNI grade to establish a nomogram for patients with postoperative TT. The C-index of the nomogram was high, and the calibration curve suggested a good match between prediction and observation for postoperative TT in both training and validation cohorts.

Patient and aneurysm characteristics could influence the consciousness of aSAH patients and severity of the disease. The elderly were considered frail when facing diseases, including sSAH. Rupture of larger aneurysms may cause greater shocks to the brain and body, as well as more severe inflammation response. Zumofen et al. reported that higher age was correlated with low GCS score, high WFNS grade, thick clot, sedation, and intubation status in aSAH patients (25). In addition, larger aneurysm size was also an independent risk factor of low GCS score, high WFNS grade, sedation, and intubation status. Therefore, age and aneurysm size are two relevant factors that determine the consciousness of aSAH patients on admission, which may later end up in TT. Humble et al. conducted a retrospective cohort study to identify risk factors related to TT placement in patients after severe traumatic brain injury (26). Their findings showed a non-linear relationship between age and TT placement. The likelihood of TT increased with the increase of age from 18 to 40, followed by a decreasing probability after reaching the peak at the age of 40. In our study, we confirmed that age ≥60 years was an independent risk factor of postoperative TT in aSAH patients. This may be caused by several reasons. First, the aSAH patients manifest a different mean age of onset from patients with severe traumatic brain injury. Second, several studies have revealed that advanced age was an independent risk factor for poor outcome of aSAH patients (27–29). The function of airway mucosal barrier, immune function, and lung compliance may weaken with age. This means that patients with advanced age tend to have a higher rate of TT due to the need of longer duration of artificial airway and respiratory support. In addition, our study revealed that larger aneurysm was strongly associated with TT in the univariable analysis but lost its significance in the multivariable analysis. This may be caused by several reasons. First, larger aneurysms may cause greater bleeding volume, which means heavier blood clot burden, and influence consciousness and severe neurological functional status. Therefore, the mFisher grade and BNI grade which accurately reflect blood clot burden and the WFNS grade may contribute to the loss of significance of aneurysm size in the multivariable analysis. Second, the other factors left may be more relevant. Third, the relatively small sample may affect the final results of multivariable analysis.

Our study investigated several inflammatory biomarkers including NLR, PLR, LMR, and SIRI. After multivariate analysis, NLR was left in the two logistic regression models with other independent factors. High NLR may reflect the more severe inflammatory state and an immune dysregulation state (elevated innate immune response and decreased adaptive immune response) of aSAH patients, which may be accompanied by several complications during the disease progression. Our study found that TT patients had a higher risk of DCVS and DCI. Notably, inflammatory biomarkers have been confirmed as an independent risk factor for several postoperative complications in aSAH patients, including TT, symptomatic vasospasm, and cerebral infarction (30, 31). However, the causal pathways and precise mechanisms of the relation between high NLR and DCVS and DCI in aSAH patients were unclear based on the current understanding. We speculated that a high inflammatory state may indicate a higher rate of DCVS and DCI and hence worse neurological outcome. Also, these factors may indicate consciences, sedation, and intubation of patients and together influence the risk of TT.

Our study also investigated the roles of some scoring scales in predicting postoperative TT, which reflected the neurological status and disease severity. Among them, WFNS grade, mFisher score, and BNI grade were left after the multivariable analysis. Previous studies have revealed that low GCS was independently associated with TT (32, 33). As the level of consciousness is a main reason for prolonged mechanical ventilation in majority of patients, GCS could be used a predictive factor for the need of postoperative TT. However, the GCS score is known to vary among physicians on admission when compared to the WFNS grade which consists of level of GCS and movement disorders. Therefore, we select WFNS grade to establish our model and confirm its significance. In addition, mFisher score and BNI grade are two important scales which could reflect the blood clot burden and severity of aSAH. Previous studies have confirmed that BNI grade was superior to mFisher in reflecting blood clot burden (18, 19). Our study confirmed that both of them could reflect the higher risk of DCVS and DCI in the TT patients. The better performance of reflecting blood clot burden may be the reason why BNI was left in the final model. In addition, a score such as SETscore consists of many terms and is not specially used to predict TT need in aSAH patients. Other scores may not comprehensively reflect the risk of TT in aSAH patients. Therefore, we thought that a score such as SETscore or others is not suitable for application in aSAH patients alone. The nomogram was guaranteed to reach an excellent predictive value based on comprehensive factors and may be more suitable for aSAH patients.

Patients with ruptured posterior circulation aneurysms usually have worse admission scores and higher mortality. Therefore, those patients may tend to have higher intubation rates on admission. However, our study did not reveal the significant association between posterior circulation aneurysms and the need of TT. On the one hand, the relatively small sample and single-center study may cause the bias of results. On the other hand, considering the high mortality of patients with posterior circulation aneurysms, many critically ill patients may die at disease onset or during interhospital transport. Those may explain why posterior circulation aneurysm was not an independent predictor for the need of TT in our study.

There were several inherent limitations applied to our study. First, as a single-center and retrospective study, insufficient sample size may introduce selection bias in the screening of subjects leading to some interference to the study. Second, the selection of surgical patients in our study may cause potential bias as some patients may refuse surgery due to severity of disease or financial hardship. Third, our study did not include all factors such as nutritional status and factors occurred during the disease course. Fourth, the validation sample was not in the same time frame as the initial training sample, which may be affected by advancements in medical practice. Further prospective studies are required to reduce the bias and confirm the application of the established nomogram and its contribution to the improvement of outcome.

## Conclusion

Our study established and validated a nomogram model for predicting postoperative TT in aSAH patients. Higher age, high NLR, high WFNS grade, and high BNI grade were proved to serve as a reliable indicator for predicting postoperative TT of aSAH patients. Further prospective studies with large sample size are required to verify our findings.

## Data Availability Statement

The raw data supporting the conclusions of this article will be made available by the authors, without undue reservation.

## Ethics Statement

The studies involving human participants were reviewed and approved by The ethics committee of the First Affiliated Hospital of Fujian Medical University. Written informed consent for participation was not required for this study in accordance with the national legislation and the institutional requirements.

## Author's Note

This work was done in Department of Neurosurgery, The First Affiliated Hospital of Fujian Medical University, Fuzhou, China.

## Author Contributions

X-YC, YC, and NL were major contributors in the concept, design, definition of intellectual content, literature search, data acquisition, data analysis, statistical analysis, manuscript preparation, manuscript editing, and manuscript review of the manuscript. J-YC and C-YD analyzed and interpreted the data. D-ZK, D-LW, and W-HF took responsibility for the integrity of the work as a whole from inception to publication of the article. All authors were involved in the research design, data analysis, drafting and critical review of the paper, and approval of the submitted version.

## Conflict of Interest

The authors declare that the research was conducted in the absence of any commercial or financial relationships that could be construed as a potential conflict of interest.

## Publisher's Note

All claims expressed in this article are solely those of the authors and do not necessarily represent those of their affiliated organizations, or those of the publisher, the editors and the reviewers. Any product that may be evaluated in this article, or claim that may be made by its manufacturer, is not guaranteed or endorsed by the publisher.
